# Elucidation of protein function using computational docking and hotspot analysis by *ClusPro* and *FTMap*

**DOI:** 10.1107/S2059798322002741

**Published:** 2022-05-25

**Authors:** George Jones, Akhil Jindal, Usman Ghani, Sergei Kotelnikov, Megan Egbert, Nasser Hashemi, Sandor Vajda, Dzmitry Padhorny, Dima Kozakov

**Affiliations:** a Department of Applied Mathematics and Statistics, Stony Brook University, Stony Brook, NY 11794, USA; b Department of Biomedical Engineering, Boston University, Boston, Massachusetts, USA; c Department of Systems Engineering, Boston University, Boston, Massachusetts, USA; d Laufer Center for Physical and Quantitative Biology, Stony Brook University, Stony Brook, NY 11794, USA

**Keywords:** protein docking, hotspots, fast Fourier transform, mapping, *FTMap*, *ClusPro*

## Abstract

Starting with a crystal structure of a macromolecule, computational structural modeling can help to understand the associated biological processes, structure and function, as well as to reduce the number of further experiments required to characterize a given molecular entity. In the past decade, two classes of powerful automated tools for investigating the binding properties of proteins have been developed: the protein–protein docking program *ClusPro* and the *FTMap* and *FTSite* programs for protein hotspot identification. These methods have been widely used by the research community by means of publicly available online servers, and models built using these automated tools have been reported in a large number of publications. Importantly, additional experimental information can be leveraged to further improve the predictive power of these approaches. Here, an overview of the methods and their biological applications is provided together with a brief interpretation of the results.

## Abstract 2

An overview of computational docking and mapping approaches, and their biological applications to the problems of drug discovery, is presented.

## 1. Introduction

X-ray crystallography provides atomistic structural details of macromolecules and is crucial for the mechanistic understanding of their cellular function. However, some applications such as drug discovery or the determination of protein–protein complexes may require further experiments and additional structures to answer all questions. In these instances, computational structural modeling tools can serve as an important alternative method to gain structural insights, as well as to guide or minimize the amount of further experiments.

This paper aims to briefly outline several state-of-the-art computational approaches that are used to help understand biological processes, structure and function, including *ClusPro*, a protein–protein docking web server, and *FTMap*, a family of web servers for determining and characterizing ligand-binding hotspots of proteins. Advanced features may be enabled to leverage pertinent *a priori* or experimental data, thereby offering more accurate predictions. Recently, *ClusPro* has been used to explore additional applications with *AlphaFold*2, including high-accuracy prediction of protein–protein interactions.

### 1.1. Protein–protein docking using *ClusPro*

*ClusPro* is a web server based on a rigid-body docking method, *PIPER*, that firstly samples all translations and rotations of a ligand protein with respect to a receptor protein and secondly uses the fast Fourier transform (FFT) correlation approach using knowledge-based or statistical potentials as the scoring function to sort the samples in order to select the best model of the complex (Kozakov *et al.*, 2006 ▸; Xia *et al.*, 2016 ▸). The server performs three computational steps as follows: (i) rigid-body docking by sampling billions of conformations, (ii) root-mean-square deviation (r.m.s.d.)-based clustering of the 1000 lowest-energy structures generated to find the largest clusters that will represent the most likely models of the complex and (iii) refinement of selected structures using energy minimization. The numerical efficiency of the method stems from the fact that such energy functions can efficiently be calculated using FFTs, which provide the ability to exhaustively sample billions of conformations of the two interacting proteins, evaluating the energies at each grid point. Thus, the FFT-based algorithm enables the docking of proteins without any *a priori* information on the structure of the complex. While *ClusPro* assumes that the proteins are essentially rigid, the method allows for moderate conformational changes due to the smoothness of the energy function and its tolerance of atomic overlaps. In fact, allowing a certain amount of overlap is key to the success of any rigid-body docking method. The resulting steric conflicts are then removed by local energy minimization of the generated complex structures. To account for larger conformational changes one can dock structures based on NMR experiments, multiple X-ray structures or structures generated by molecular-dynamics (MD) simulations. In spite of these approaches, we admit that without access to multiple representative structures, docking proteins that substantially alter their conformation upon binding is a difficult and not entirely solved problem.

In some cases one has additional experimental information on the complexes such as cross-linking (XL-MS) or mutational data, which can offer information regarding pairs of atoms or residues at a protein interface. Such information can be used to generate pairwise distance restraints that can be provided as input to *ClusPro*. If interface restraints are available then only portions of conformational space will be examined by the program (Xia *et al.*, 2016 ▸); thus, the restraints provide more reliable predicted structures using the *ClusPro* scoring function and also reduce the computational cost. Furthermore, the confidence in the restraints can be modified by changing the number of restraints to be satisfied during the *PIPER* docking process.

The *ClusPro* docking methodology has consistently been the top-performing server in Critical Assessment of Predicted Interactions (CAPRI; Lensink *et al.*, 2007 ▸, 2019 ▸; Lensink & Wodak, 2010 ▸, 2013 ▸), a double-blinded protein–protein docking experiment. The *ClusPro* server has more than 20 000 registered academic users and has performed more than 600 000 jobs in the last ten years.

### 1.2. Ligand-binding site determination and characterization with *FTMap*

Given the protein crystal structure, a number of questions can be posed in the context of drug discovery. Some of these questions are as follows. What are the functional binding sites of the protein? Can the site of important biological function be targeted by high-affinity small molecules (*i.e.* is the pocket druggable)? Given the binding site how can a ligand be most optimally designed, or given a natural ligand how should it be modified or extended? Here we describe a computational solvent-mapping algorithm, *FTMap*, which provides answers to these questions (Kozakov *et al.*, 2015 ▸). Requiring only a protein, DNA or RNA structure in PDB format as input, *FTMap* samples millions of positions of small organic molecules used as probes and scores the probe poses using a detailed molecular-mechanics-like energy expression. *FTMap* has been developed as a close computational analog of screening experiments based on X-ray crystallography (Mattos & Ringe, 1996 ▸) or NMR (Hajduk *et al.*, 2005 ▸). The method distributes small organic probe molecules of varying size, shape and polarity on a macromolecule surface, finds the most favorable positions for each probe type and then clusters the probes and ranks the clusters on the basis of their average energy. These probes include 16 organic molecules (ethanol, 2-propanol, isobutanol, acetone, acetaldehyde, dimethyl ether, cyclohexane, ethane, acetonitrile, urea, methylamine, phenol, benzaldehyde, benzene, acetamide and *N*,*N*-dimethylformamide). Furthermore, regions that bind several probe clusters are referred to as consensus sites and define binding hotspots that substantially contribute to the binding free energy. Analogous to experiments, the larger the probe population at a particular site the more important the hotspot is. The number of probe clusters forming a consensus site is strongly correlated with ‘druggability’ and the relative importance of the site. The hotspots can be further combined to identify protein binding sites. This approach is performed by *FTSite* (Ngan *et al.*, 2012 ▸), which builds on top of *FTMap*. The mapping process used by *FTMap* and *FTSite* can take into account small conformational changes for the reasons described above for *ClusPro*. Additionally, hotspots tend to be conserved despite moderate conformational changes (Kozakov *et al.*, 2011 ▸). Large conformational changes can be explored by applying *FTMap* to ensembles of structures generated either by NMR, MD or multiple crystal structures using an MD ensemble.

## 2. Results

### 2.1. Protein–protein docking using *ClusPro*

Two protein–protein docking applications are presented here. The first is *ab initio* docking and the second is docking guided by experimental restraints.

#### 2.1.1. *Ab initio* protein–protein docking

**Figure 1:**
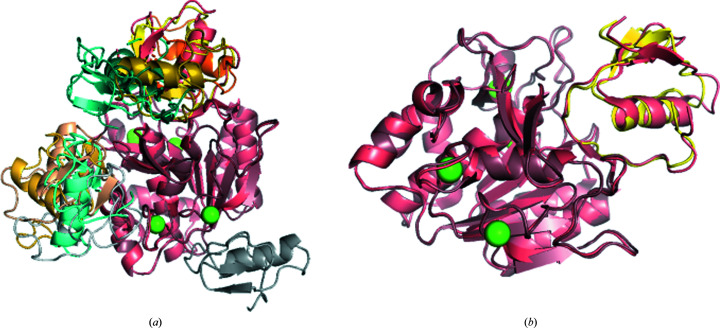
Protein–protein docking using *ClusPro*. (*a*) *ClusPro* produces multiple models of the ligand (PDB entry 2gkr) binding to the receptor (PDB entry 1scn). The top ten models using the balanced coefficient set are presented. (*b*) The PDB entry 1r0r structure is shown in salmon, the PDB entry 1scn structure is shown in brown and the number 2 ranked ligand (PDB entry 2gkr) is shown in yellow.

Here, we demonstrate a case of protein–protein docking starting from separately crystallized subunits. As an example, we consider a complex of subtilisin Carlsberg protease (PDB entry 1scn) and its inhibitor turkey ovomucoid third domain (OMTKY3; PDB entry 2gkr). The unbound structures, PDB entries 1scn and 2gkr, are submitted to *ClusPro* without any additional information. The top ten results of this docking run are shown in Fig. 1 ▸(*a*) superimposed onto an X-ray structure of the complex (PDB entry 1r0r). In Fig. 1 ▸(*b*) the near-native *ClusPro* model ranked 2 is highlighted. The model provides a reasonable approximation of the binding found in the crystal structure (PDB entry 1r0r) and shows an r.m.s.d. of 2.09 Å to the native structure.

#### 2.1.2. Protein–protein docking with distance restraints

**Figure 2:**
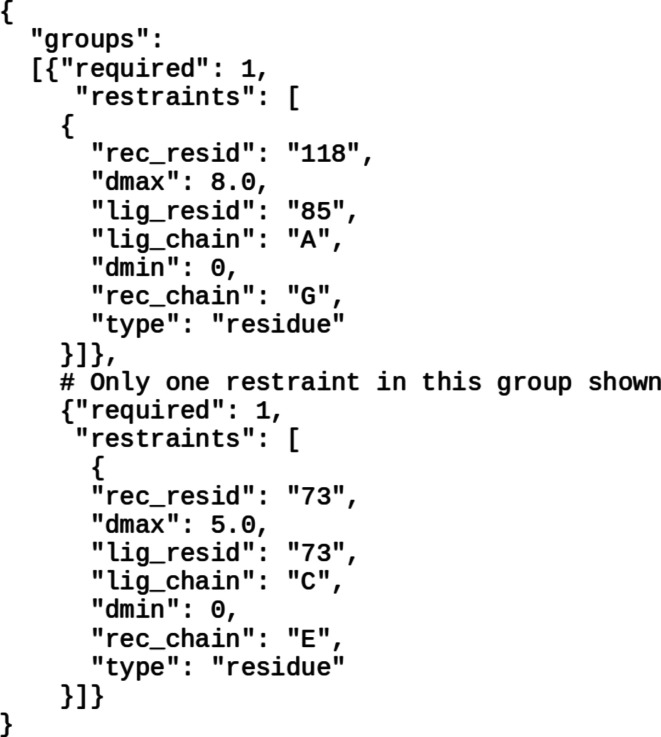
Restraint formatting. The figure illustrates the format of the restraints used for this docking option.

**Figure 3:**
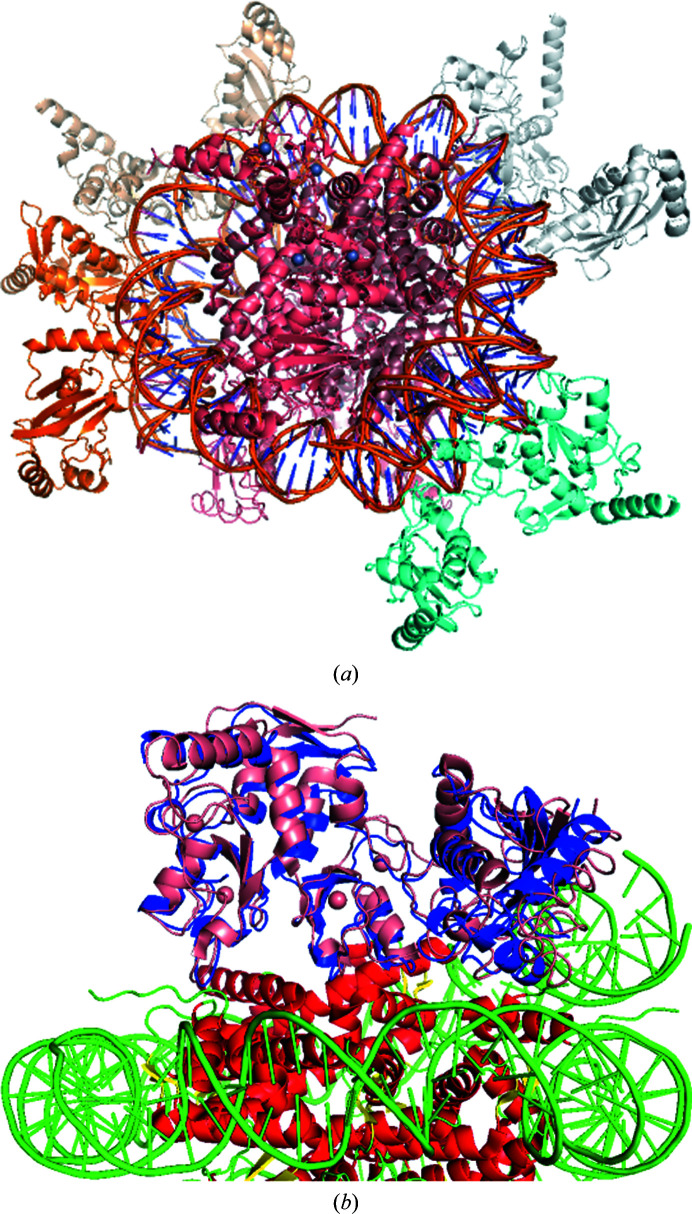
Protein–protein docking with restraints. Docking results using *ClusPro*, both restrained and unrestrained. (*a*) The unrestrained docking results for the Bmi1/Ring1b–UbcH5c complex and nucleosome. The Bmi1/Ring1b–UbcH5c complex is bound to the DNA in this instance. (*b*) This is the number 2 ranked pose using restraints; it binds to the appropriate location and has a near-native pose.

To demonstrate docking with experimental restraints we consider the case of the Bmi1/Ring1b–UbcH5c complex (PDB entry 3rpg) binding to a nucleosome core particle (PDB entry 3lz0). When the docking run is submitted without the use of restraints the Bmi1/Ring1b–UbcH5c complex is modeled as binding to the DNA strand, which contradicts experimental evidence. The ubiquitination process indicates that the Cys85 residue on UbcH5c needs to be proximal to the Lys119 residue on H2A of the nucleosome (Bentley *et al.*, 2011 ▸). There are also mutational studies which indicate that Lys97 on Ring1b is involved in binding to the surface of the core histones (Bentley *et al.*, 2011 ▸). These experimental details can be used to specify geometric restraints which will limit the search space to the relevant areas. The generation of restraints can be performed using the restraint generator provided at https://cluspro.bu.edu/generate_restraints.html. The generator outputs a restraint file formatted as shown in Fig. 2 ▸. The results of the restrained docking can be viewed in Fig. 3 ▸(*b*) compared with the crystal structure of the complex found in PDB entry 4r8p. This can be compared with the unrestrained docking results shown in Fig. 3 ▸(*a*). The restrained results provide a binding pose close to the reported structure among the top predictions: this is the pose ranked 2 and it has an iRMSD of 4.9 Å (see Fig. 3 ▸ *b*).

### 2.2. Identification of ligand-binding hotspots using *FTMap*

In this section, we demonstrate hotspot identification using *FTMap* in various drug discovery-related applications starting from the crystal structure of the protein.

#### 2.2.1. Fragment screening for SARS-CoV-2 main protease with *FTMap*

**Figure 4:**
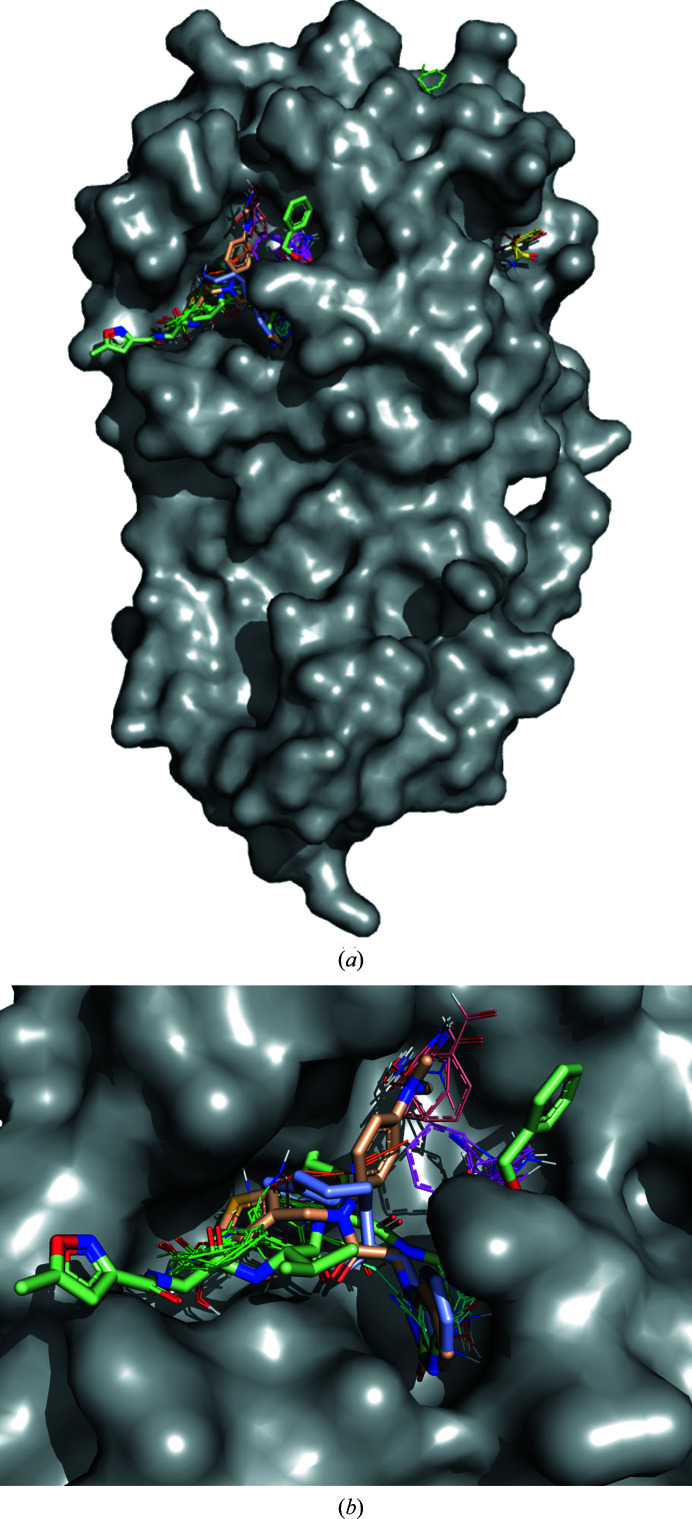
Fragment screening for Mpro using *FTMap*: the top-ranking consensus clusters of probes are depicted in green, cyan, magenta and yellow. The SARS-CoV-2 Mpro protein structure is depicted as a gray surface in a global view (*a*) and the active site (*b*). The inhibitors are peptide-like (pale green sticks; Jin *et al.*, 2020 ▸), Diamond Fragalysis (wheat sticks; XChem@Diamond; https://fragalysis.diamond.ac.uk/viewer/react/landing) and PostEra COVID Moonshot (light blue sticks; https://postera.ai/moonshot).

As a first example of computational binding-site prediction with *FTMap*, we applied *FTMap* to SARS-CoV-2 main protease (Mpro; Douangamath *et al.*, 2020 ▸), a recognized COVID-19 drug target. Fig. 4 ▸(*a*) demonstrates the global mapping of Mpro shown in a gray surface representation. *FTMap* produced nine consensus sites or hotspots ranked by cluster population and shown as different carbon-color line representations. There are four mostly minor consensus sites outside the active site of Mpro, including two near the dimerization interface. The majority (4/5) of highly populated consensus sites with over ten probe clusters can be found in the active site of Mpro, including the consensus site with the highest population (26 probe clusters), which implies that the site is druggable. Indeed, to date, several compounds with submicromolar binding to Mpro have been reported in the literature. Enlarging the active site shown in Fig. 4 ▸(*b*), one can see that the compounds depicted in stick representation overlap with *FTMap* hotspots in different combinations.

#### 2.2.2. Druggability analysis of protein–protein interfaces using *FTMap*

**Figure 5:**
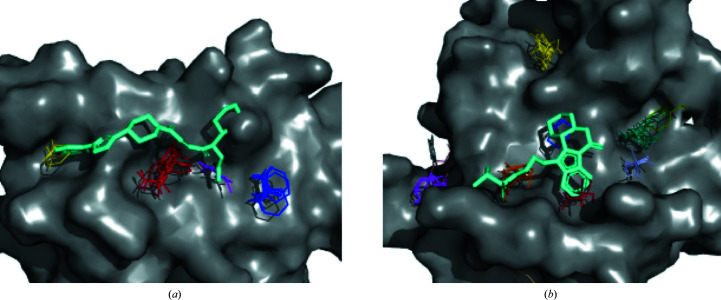
Protein–protein interface druggability. Druggability analysis of relevant protein–protein interfaces using *FTMap*. (*a*) *FTMap*-generated hotspots at the interface of interleukin-2 (PDB entry 1m47) with the interleukin-2 receptor and the small-molecule inhibitor FRB (PDB entry 1pw6; IC_50_ = 6 µ*M*). Clusters 1 (red, 18 probes), 4 (blue, 12 probes) and 9 (magenta, three probes) constitute a druggable site at the interface. Moreover, clusters 1, 4 and 7 (yellow, five probes) are in close proximity to the inhibitor. (*b*) *FTMap*-generated hotspots at the interface of ZipA (PDB entry 1f46) with FtsZ and the weak small-molecule inhibitor WAC (PDB entry 1s1s). There were no strong hotspots at the interface to form a druggable site. The inhibitor is in close proximity to the low-strength clusters 5 (orange, eight probes), 10 (red, three probes) and 13 (blue, two probes). The low binding affinity of the inhibitor at the interface is consistent with the *FTMap* prediction of the interface not being druggable

The low druggability of protein–protein interfaces for the binding of drug-like small molecules is a grand challenge in drug discovery. It is especially difficult due to the relatively shallow pockets on the protein surface compared with those found in traditional protein–ligand interactions, and the requirement of the ligand to compete with protein interactions. *FTMap* can be used to identify ‘hotspots’ on the protein surface, the presence, strength and relative distance of which on the interface can indicate druggable sites. Fig. 5 ▸(*a*) highlights the *FTMap* results of mapping interleukin-2 at its interface with the interleukin-2 receptor. There are strong hotspots present (≥16 probes) along with other hotspots that indicate a druggable site. Indeed, low-nanomolar inhibitors were found for this interface. Fig. 5 ▸(*b*) highlights the contrasting results for ZipA at its interface with FtsZ, where although some hotspots are present they are weak and do not indicate a druggable site. In fact, only weak ligands were found for this interface, which supports the prediction.

#### 2.2.3. Identifying allosteric sites using *FTMap*

**Figure 6:**
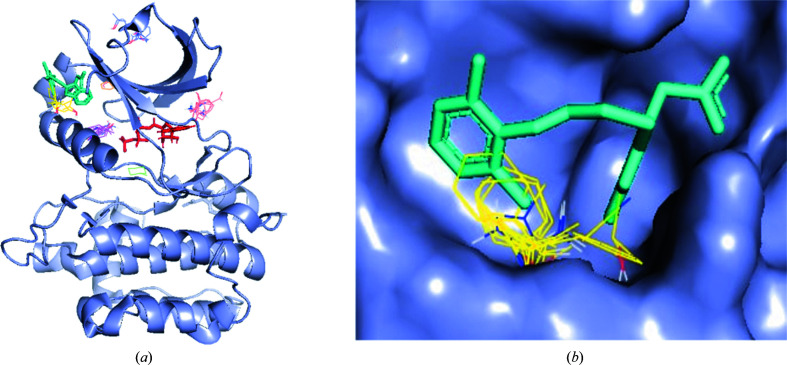
Protein mapping using *FTMap*. (*a*) The *FTMap* results for the N lobe of PDB entry 1h1w, with the PIF pocket in yellow, the ATP-binding pocket in magenta, the ATP molecule in red and adenosine in teal. (*b*) Mapping of the PIF binding pocket (yellow) with the bound ligand RF4 (teal).

Targeting allosteric sites on kinases is an emerging area in drug discovery. Since *FTMap* searches for sites on the entire protein surface, it can be useful for finding such sites. Here, we demonstrate the application of the approach to the identification of allosteric sites on PDK1 kinase. The kinase example is also interesting since kinases are multi-domain proteins and *FTMap* was optimized to work on single domains. To address this, in addition to mapping the entire protein (PDB entry 1h1w) we separately map the domains (N and C lobes in this case). These two lobes are then submitted to *FTMap*. PDK1 binds ATP in its main pocket; in addition, an allosteric regulation site has also been identified, the PDK1-interacting fragment (PIF) pocket. The mapping results for the N lobe are located in Fig. 6 ▸(*a*), and the two most populated identified pockets, corresponding to the ATP-binding site and the PIF site, are shown in Fig. 6 ▸(*b*) along with a bound ligand (PDB entry 4xx9). Application of *FTMap* in the analysis to identify cryptic and allosteric sites is discussed in more detail in Beglov *et al.* (2018 ▸). Analysis of structures in the kinome are provided in Yueh *et al.* (2019 ▸).

#### 2.2.4. Detection of ligand-binding sites using *FTSite*

**Figure 7:**
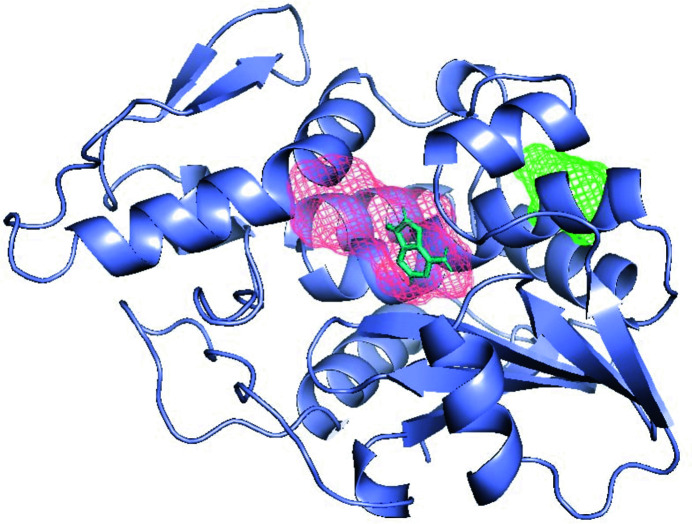
Protein mapping using *FTSite*. The *FTSite* results for PDB entry 1ahc shown with the bound ligand adenosine (teal). The first predicted pocket (red) and the second predicted pocket (green) are shown.

Nearby hotspots predicted by *FTMap* can be further combined to predict entire binding sites. This is performed by the *FTSite* algorithm available as part of the *FTMap* family of servers. We demonstrate binding-site identification of the ribosome-inactivating protein (RIP) momordin. The protein is known to bind adenosine. We predict the binding site of the protein starting with unliganded momordin (PDB entry 1ahc). The top two pockets predicted by *FTSite* are shown in Fig. 7 ▸ along with the ligand overlapped from the bound structure (PDB entry 1mrg). The adenosine pose lies within the first-ranked pocket.

### 2.3. Docking and mapping using high-accuracy protein models

*AlphaFold*2 has made landmark advances in protein structure prediction (Jumper *et al.*, 2021 ▸). Here, we present several applications of high-accuracy protein models to predict both protein–protein interactions (PPI) using *ClusPro* and ligand-binding sites using *FTMap*.

#### 2.3.1. Predicting protein–protein interactions with *AlphaFold*2 and *ClusPro*

**Figure 8:**
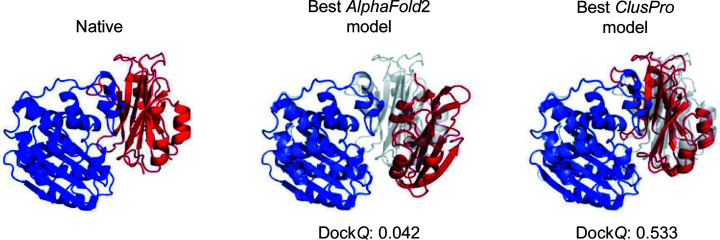
Docking comparison between *AlphaFold*2 and *ClusPro*. Docking results for β-lactamase inhibitory protein (UniProt P35804) and the β-lactamase TEM1 (UniProt P62593) from the protein–protein docking benchmark (Vreven *et al.*, 2015 ▸). Comparison of the best docked models produced by *AlphaFold*2 and those produced by the docking of *AlphaFold*2 subunits using *ClusPro*.

Firstly, we demonstrate the docking of models of individual protein monomers using *ClusPro*. We consider the complex between the β-lactamase inhibitory protein and β-lactamase as an example, and construct the monomer models using* AlphaFold*2. The sequences of the component proteins are those of the unbound structures in the PDB. We then used the *MMseqs*2 API to generate multiple sequence alignments (MSA) for each sequence, which were then combined (Mirdita *et al.*, 2019 ▸). In order to allow generation of the complex, we introduced a 200-residue gap in the residue-index numbering between each protein. We used the pTM model parameter set to generate models using *AlphaFold*2. *AlphaFold*2 provided a predicted aligned error (PAE) for each residue of the model, which we used to calculate an average PAE score for those residues at the interface of the interacting proteins. The interface was defined to be those residues that were within 10 Å of the other protein. The *AlphaFold*2 model of the complex with the lowest average interface PAE score was selected and split into two separate structures representing the receptor and the ligand. As can be seen from Fig. 8 ▸, *AlphaFold*2 was not able to generate an accurate protein complex in this case. However, when we provided the monomer model to *ClusPro* for docking, it was able to generate a high-accuracy model of the complex.

#### 2.3.2. Predicting binding sites with *AlphaFold*2 and *FTMap*

**Figure 9:**
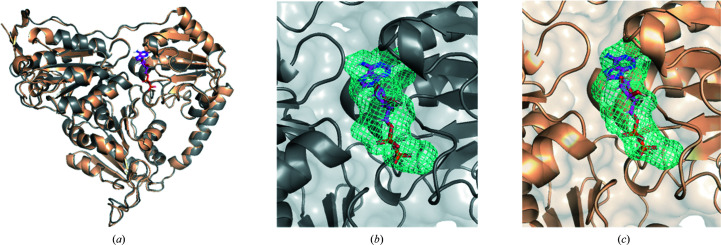
The *AlphaFold*2 prediction of HACL bound to ADP is indistinguishable from the X-ray structure. (*a*) The X-ray structure, PDB entry 6xn8, is shown as a gray cartoon with co-crystallized ADP shown as pink sticks. The top *AlphaFold*2 model is overlaid in wheat (GDT_TS = 99.07). (*b*) The *FTMap*-predicted ADP-binding site in the X-ray structure. (*c*) The *FTMap*-predicted ADP-binding site in the top *AlphaFold*2 model, with PDB entry 6xn8 chain *A* with ADP overlaid as a reference.

Similar to the case of protein docking, accurate models of proteins can be used with *FTMap* to perform the mapping of predicted binding sites on protein surfaces. The binding properties of high-quality protein models produced by *AlphaFold*2 (generally GDT_TS > 90) have been shown to correlate with the binding properties of experimental structures in the functional analysis of CASP14 targets (Egbert *et al.*, 2021 ▸). For example, the protein 2-hydroxyacyl-CoA lyase (HACL) was co-crystallized with ADP bound and was utilized as a CASP14 target. The model predicted by *AlphaFold*2 is almost an exact match (GDT_TS = 99.07) to the X-ray structure, and the ADP-binding pockets are nearly identical (see Fig. 9 ▸). *FTMap* of both the X-ray structure and the *AlphaFold*2 prediction identified the ADP-binding site as the strongest site, with 33 and 26 probe clusters, respectively. Visually, the predicted binding sites in HACL appear to be almost identical between the X-ray structure and the *AlphaFold*2 model.

## 3. Conclusions

In this work, we show various applications of computational docking using *ClusPro* and hotspot identification using *FTMap*. Both servers use protein crystal structures as inputs. We demonstrate that *ClusPro* can be used to predict high-accuracy models of protein complex structures with and without the use of experimental information. *FTMap* enables the identification of orthosteric and allosteric binding sites in proteins, determining the druggability (*i.e.* the ability to develop high-affinity small molecules) of sites of biological interest and also provides information for the design of small-molecular inhibitors and modulators. We demonstrate that the tools can also be used with high-accuracy protein models provided by novel deep-learning algorithms such as *AlphaFold*2. The methods are available for free to academic users by means of public web servers. All of the input models for the <!?tlsb=-.017w>server are available at https://cluspro.bu.edu/examples/inputs.zip.
